# An efficient algorithm to compute marginal posterior genotype probabilities for every member of a pedigree with loops

**DOI:** 10.1186/1297-9686-41-52

**Published:** 2009-12-03

**Authors:** Liviu R Totir, Rohan L Fernando, Joseph Abraham

**Affiliations:** 1 Pioneer Hi-Bred International, A Dupont Business, 7250 NW 62nd Ave, Johnston, Iowa 5013, USA; 2 Department of Animal Science and Center for Integrated Animal Genomics, Iowa State University, Ames, Iowa 50011, USA; 3 Case Western Reserve University, Cleveland, Ohio 44106, USA

## Abstract

**Background:**

Marginal posterior genotype probabilities need to be computed for genetic analyses such as geneticcounseling in humans and selective breeding in animal and plant species.

**Methods:**

In this paper, we describe a peeling based, deterministic, exact algorithm to compute efficiently genotype probabilities for every member of a pedigree with loops without recourse to junction-tree methods from graph theory. The efficiency in computing the likelihood by peeling comes from storing intermediate results in multidimensional tables called cutsets. Computing marginal genotype probabilities for individual *i *requires recomputing the likelihood for each of the possible genotypes of individual *i*. This can be done efficiently by storing intermediate results in two types of cutsets called anterior and posterior cutsets and reusing these intermediate results to compute the likelihood.

**Examples:**

A small example is used to illustrate the theoretical concepts discussed in this paper, and marginal genotype probabilities are computed at a monogenic disease locus for every member in a real cattle pedigree.

## Background

For monogenic or oligogenic traits, algorithms for efficient likelihood computations have been described for both pedigrees without loops [1], and pedigrees with loops [2-5] Furthermore, efficient algorithms have been developed to draw samples from the joint posterior distribution of genotypes from complex pedigrees [6,7]. However, when pedigrees are large with many loops and multiple loci, these sampling methods can become very inefficient, and the J-PCS algorithm was proposed to address this problem [8]. This algorithm involves a) modifying the pedigree by cutting some loops and b) sampling the genotype of an individual *i *that is as distant as possible from the modifications ("cuts"). This sample must be drawn from the marginal posterior genotype probability distribution of *i *given the modified pedigree, which may still have many loops. Furthermore, marginal posterior genotype probabilities are needed in genetic counseling in humans and selective breeding in domesticated species. An efficient, exact, deterministic algorithm is available to compute the marginal posterior genotype probabilities for every member in a pedigree without loops [9]. However, it is not straightforward how to extend this algorithm to compute marginal posterior genotype probabilities for pedigrees with loops. Recently, junction tree methods from graph theory were used to describe an efficient algorithm to compute marginal posterior genotype probabilities for pedigrees with loops [10]. Most geneticists, however, are not familiar with junction tree concepts, and thus such algorithms would not readily be incorporated in genetic analyses, especially because the paper of Lauritzen and Sheehan [10] is not self-contained, but relies on results from other sources. In this paper, we present a self-contained description of an efficient, exact, deterministic algorithm to compute marginal posterior genotype probabilities for every member of a pedigree with loops, without use of junction tree methods. This algorithm has been implemented in the public domain software package MATVEC and can be obtained from the corresponding author.

Following is a brief outline of the presentation. First we define pedigree loops. Next we discuss the relationship between the likelihood and marginal posterior genotype probabilities of pedigree members. Following this, anterior and posterior cutsets are introduced. Anterior cutsets are used to compute the likelihood by the Elston-Stewart algorithm (peeling), and anterior and posterior cutsets are used to describe an efficient algorithm to calculate marginal probabilities for every member of a pedigree with loops. Next, marginal genotype probabilities are calculated for every member in a cattle pedigree that contains loops. Finally, in the appendix, a small example is used to illustrate in detail the theoretical concepts discussed in this article.

## Methods

### Definition of Pedigree Loops

Here we define pedigree loops indirectly by providing a simple algorithm to determine if a pedigree contains loops. A pedigree is a set of individuals, each of which can be classified as a founder or a non-founder. A founder is a pedigree member whose parents are not in the pedigree, and a non-founder is a pedigree member with both parents present in the pedigree. A nuclear family consists of a set of parents and all their off spring. A terminal family is a family that has at most one member who belongs to at least one other nuclear family. Terminal members of a pedigree are members of terminal families that do not belong to another family. The algorithm used to determine if a pedigree contains loops relies on identifying and then eliminating terminal members from the pedigree. If a pedigree does not contain any loops, repeated removal of terminal members from the pedigree will result in all members being removed from the pedigree. On the other hand, if a pedigree contains any loops, not all members of the pedigree can be removed by repeated removal of terminal members. See additional file 1: "Algorithm to detect loops.pdf" for an example of the use of this algorithm to identify loops in arbitrary pedigrees.

### Likelihood and Genotype Probability Calculations for General Pedigrees

Consider a pedigree with *n *individuals, and let *g*_*i *_denote the possible genotype and *y*_*i *_the observed phenotype of an arbitrary pedigree member *i*. Note that both *g*_*i *_and *y*_*i *_can be a function of a single locus or of multiple loci on the chromosome. The likelihood for a genetic model given the observed data can be written as(1)

where *F*(***g***, ***y***; *ρ*, ***q***, ***θ***) denotes the joint distribution of all *g*_*i *_(***g***) and all *y*_*i *_(***y***) in the pedigree, ***ρ ***is the vector of recombination rates between loci, ***q ***is the vector of gene frequencies, and ***θ ***is the vector of parameters in the genetic model that relates *y*_*i *_and *g*_*i *_[11]. Furthermore, the likelihood can be written as(2)

where  is a set of possible genotypes of a given set of pedigree members *s*_*i*_, and  is defined as(3)

where *h*(*y*_*i*_| *g*_*i*_, ***θ***) is the conditional probability of the phenotype *y*_*i *_given the genotype *g*_*i *_(also known as the penetrance function of individual *i*), Pr(*g*_*i*_| ***q***) is the marginal probability that a founder has genotype *g*_*i *_(founder probability) and Pr(*g*_*i*_| , , ***ρ***) is the probability that a non-founder has genotype *g*_*i *_given that its mother (*m*_*i*_) has genotype  and its father (*f*_*i*_) has genotype  (transition probability). When *g*_*i*_,  and  consist of multiple loci, the multilocus transition probability can be written as a product of single-locus transition probabilities and recombination probabilities between adjacent loci, by making use of the Markov property for recombination events between adjacent loci that holds under the assumption of no interference [5,12]. Note that, for each individual *i *in the pedigree, a set *s*_*i *_is defined that contains either one or three individuals. For founders, *s*_*i *_contains only *i*, while for non-founders, *s*_*i *_contains *i, m*_*i *_and *f*_*i*_. For an arbitrary pedigree member *i*, marginal genotype probabilities can be written as(4)

where *L *is the likelihood defined in 2, and  is the likelihood computed with *g*_*i *_fixed at genotype *x*. Thus, the efficient computation of marginal genotype probabilities using equation 4 requires an efficient algorithm to compute the likelihood. The computation of the likelihood using 2 is not efficient for pedigrees having more than about 20 members. However, the Elston-Stewart algorithm, which is also known as peeling, can be used to efficiently compute the likelihood [1,13]. Still, using equation 4 to compute marginal probabilities for *N *unknown genotypes of individual *i *requires recomputing the likelihood with *g*_*i *_= *x *for each of the *N *values of *x*. Furthermore, this has to be repeated for all *n *individuals in the pedigree. In the following section we introduce an algorithm to avoid repeating computations by storing intermediate results in multidimensional tables called anterior and posterior cutsets.

### Anterior and Posterior Cutsets

Computing the likelihood by peeling involves summing over the genotypes of one individual at a time and storing the intermediate results. For convenience, here we assume that individuals are numbered in the order that they are peeled. Peeling the first individual amounts to computing the sum over *g*_1 _of the product of all factors in 2 that contain *g*_1_, for each combination of the other genotypes that occur together with *g*_1_. Results of these summations are stored in a multidimensional table that has been called a cutset [13]. Here we will refer to these tables as anterior cutsets. The anterior cutset obtained after peeling *g*_1 _will be denoted by  and is calculated as(5)

where *V*_1 _is a set of pedigree members defined as follows. Using the sets *s*_*i *_defined earlier for each individual in the pedigree, *U*_1 _is defined as the union of all *s*_*j *_that contain individual 1. Then *V*_1 _is obtained by removing individual 1 from *U*_1_. Further,  is the set of genotypes for the individuals in *V*_1_. Note that the product in 5 is over those pedigree members *j *that contain individual 1 in their *s*_*j*_.

Replacing in 2 the product of all factors containing *g*_1_, summed over *g*_1_, with  gives the following expression for the likelihood(6)

where ***g***_1 _= {*g*_2 _... *g*_*n*_} is the set of possible genotypes of the individuals that remain to be peeled, and the product is over those pedigree members *r *that do not contain individual 1 in their *s*_*r*_. The likelihood expressed as above after peeling *g*_1_, will be referred to as *LE*_1_, and in general after peeling *g*_*i*_, will be referred as *LE*_*i*_.

Note that after *g*_1 _has been peeled, the summation in 6 is only over the genotypes of individuals 2 ... *n*. As described below, and later illustrated through a hypothetical example in the Appendix, as each individual is peeled, an anterior cutset is generated. After peeling the last individual, the final anterior cutset will have only a single value that is equal to the likelihood. Note that for a pedigree with *n *members, there are *n*! possible peeling orders. Although any choice of a peeling sequence will lead to the same value for the likelihood, not all choices of the peeling sequence lead to anterior cutsets of the same size. As the amount of memory required does depend on the size of the cutsets, a peeling sequence leading to smaller cutsets is more desirable. However, even for moderately large *n*, an exhaustive search for an efficient peeling sequence is not feasible. Furthermore, there is no known algorithm to efficiently find the peeling order with the lowest storage requirements [10]. However, the following simple heuristic procedure can be used to generate a good peeling sequence. At any stage of the peeling process, in order to decide which individual should be peeled next, for each individual *i *that remains to be peeled, we compute the size of the anterior cutset that would be generated by peeling *i*. The individual with the smallest anterior cutset size is chosen to be peeled next [14].

Now it is convenient to introduce the posterior cutset which will be used to avoid repeating computations in calculating genotype probabilities. By factoring out  from 6 and by summing over the genotypes of all remaining pedigree members not contained in *V*_1_, we can define a second multidimensional table called a posterior cutset(7)

where  is not a function of *g*_1_. As a result we can rewrite the likelihood as follows(8)

In the general description of peeling given below, we make extensive use of two sets defined for each individual *i*. The first set *s*_*i *_has already been described earlier, and it is completely determined by the pedigree. The second set *V*_*i *_contains the individuals in the cutset that is generated when *i *is peeled. Thus, *V*_*i *_is determined by the pedigree and the peeling order. In general, peeling individual *i *amounts to computing the sum over *g*_*i *_of the product of all factors in *LE*_*i*-1 _that contain *g*_*i*_, for each combination of the other genotypes that occur together with *g*_*i*_. These summations are stored in the anterior cutset for *i*:(9)

where *j *is an individual whose function *f*_*j*_() remains in *LE*_*i*-1 _and *i *∈ *s*_*j*_, *k *is an individual whose anterior cutset  remains in *LE*_*i*-1 _and *i *∈ *V*_*k*_, *U*_*i *_= () ∪ (∪ *V*_*k*_), and *V*_*i *_= *U*_*i*_-*i*. Replacing in *LE*_*i*-1 _the sum over *g*_*i *_of the product of all factors containing *g*_*i *_with  gives the likelihood expression *LE*_*i*_:(10)

where  are the functions from *LE*_*i*-1 _that were not used in the calculation of  and  are the anterior cutsets from *LE*_*i*-1 _that were not used in the calculation of . Now we obtain the posterior cutset for *i *by removing  from *LE*_*i*_:(11)

Note that  is not a function of *g*_*i*_. Thus, in general we can write the likelihood as follows(12)

Now we are ready to explain how to compute genotype probabilities for any individual *m *∈ *V*_*i *_using anterior and posterior cutsets. As in equation 4, marginal genotype probabilities for *m *can be written as(13)

The denominator of 13 is given by 12, while the numerator is obtained by computing 12 with *g*_*m *_fixed at *x*. If *m *is in more than one set of pedigree members *V*_*i*_, identifying the set *V*_*i *_with smallest number of members will minimize the required computations. However, if *m *is not in any *V*_*i*_, we first write the likelihood 12 as a product of the anterior and posterior cutsets for *m*. In this expression, however, *m *has already been peeled. Equation 9, which is used to compute the anterior cutset for an arbitrary individual, contains that individual prior to it being peeled. Thus, by substituting in 12, the expression given in 9 for  gives(14)

Now the numerator of 13 is obtained by computing 14 with *g*_*m *_fixed at *x*.

Provided a good peeling sequence is available, computation of the required anterior cutsets and the summation over  in 12 or  in 14 would be feasible. However, posterior cutsets cannot be computed efficiently using 11 because here the summation may be over a very large set of genotypes. Fortunately, posterior cutsets can be computed recursively as described below. Although the derivation of the recursive algorithm given below is conceptually straightforward, it may be tedious to follow. Thus, at the end of this section, we provide four easy to implement steps to efficiently compute posterior cutsets.

The key principle that we have used to compute marginal posterior probabilities efficiently is that any pedigree member can be assigned into one of three mutually exclusive sets with respect to any individual *i*: the set of members that contribute to , the set of members that contribute to , or the set of members in *V*_*i*_. For example, in computing the numerator of 13 by using 12, the intermediate results from peeling individuals in the first set were stored in  and used repeatedly, the intermediate results from peeling individuals in the second set were stored in  and used repeatedly, and only the calculations for peeling individuals in the third set were repeated. This principle of factoring the likelihood into anterior and posterior components is used repeatedly in the following derivations. To derive the recursive algorithm, first we establish that  = 1.0, which is the base case of the recursion. Similar to 10, after peeling individual *n *- 1, the likelihood expression *LE*_*n*-1 _becomes(15)

Because only individual *n *remains to be peeled, *V*_*u *_and *V*_*n*-1 _contain only *n*. The likelihood now becomes(16)

Further, using 9,  can be written as(17)

Note that in 16 and 17 the right-hand sides are identical, and thus *L *= . However, from 12(18)

and thus  = 1.0. Now, for any other individual *i*,  can be computed recursively as follows.

The anterior cutset  generated when *i *is peeled, is used in the calculation of the anterior cutset generated when *k *= min(*V*_*i*_) is peeled. The resulting anterior cutset can be written as(19)

where  are all remaining functions with *k *∈ *s*_*r*_, and  are the remaining anterior cutsets with *k *∈ *V*_*j *_in addition to . Similar to (12) we can also write(20)

and by using (19) in (20) we can write(21)

Recall that we have defined the set of individuals *U*_*k *_= *V*_*k*_∪ {*k*}, and thus we can write(22)

Note that both (12) and (22) contain the term . By rearranging 22, the likelihood can be written as(23)

and using 12 we can write(24)

Thus, the posterior cutset for individual *i *can be expressed as a function of some anterior cutsets and the posterior cutset for individual *k *>*i*. Starting at individual *n *- 1 all posterior cutsets can be computed in the reverse order of peeling because  = 1.0.

In summary, the following procedure can be used to recursively compute the posterior cutset of an arbitrary individual *i *in a pedigree:

1. Compute anterior cutsets for all individuals in the pedigree. This step is done only once.

2. Identify the anterior cutset  whose summand contains the factor  (see equation 19).

3. Replace  in the summand of  with , and for each value of  sum over the remaining genotypes in this expression (see equation 24).

4. If  has not been computed yet, use steps 2, 3 and 4 to compute it (this is the recursion).

Note that to compute marginal posterior genotype probabilities for an arbitrary member of the pedigree using this algorithm, we need to calculate all anterior cutsets and a subset of all posterior cutsets. Both the anterior and the posterior cutset of a given individual have the same size. The computation of an anterior cutset involves the summation over the genotypes of one individual. The computation of a posterior cutset can involve summations over the genotypes of a variable number individuals. The theoretical concepts introduced in this section are illustrated in detail for a simple example in the Appendix. In the following section we discuss a real data application of the theoretical concepts described above.

### Genotype Probabilities Computations in a Real Cattle Pedigree

**Figure 1:**
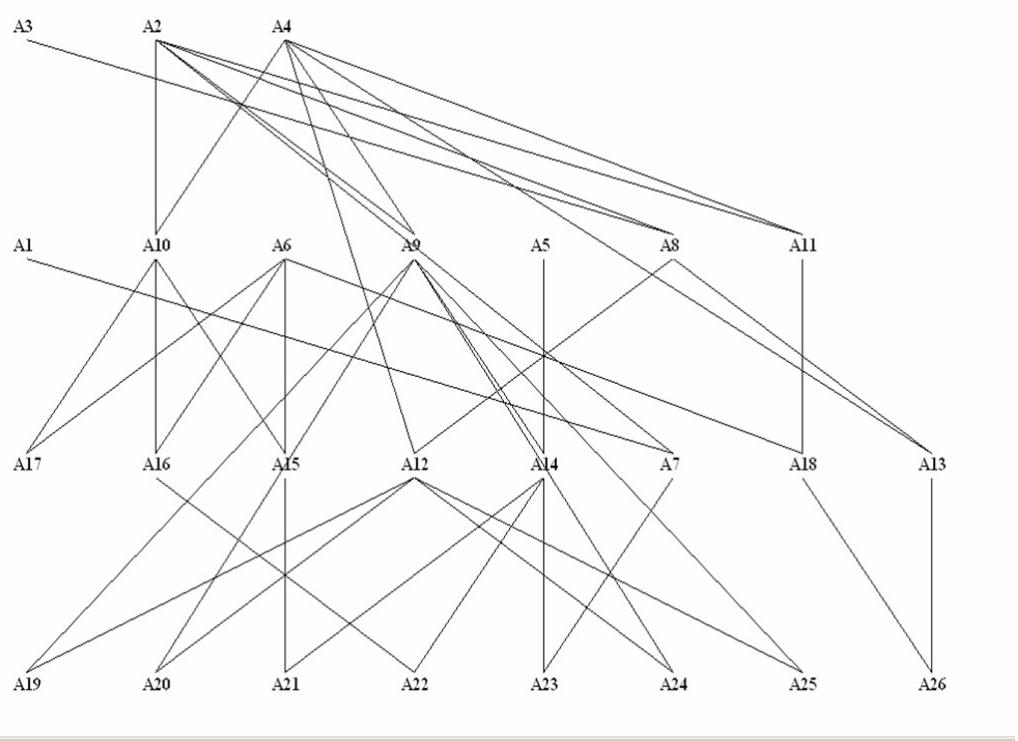
**Real example pedigree**.

Consider the pedigree given in the first three columns of Table 1 with a graphical representation given in Figure 1. Six terminal members of this cattle pedigree (individuals A21, A22, A23, A24, A25 and A26) are known to be affected by a monogenic recessive disease. Conditional on disease status, assumed mode of inheritance, pedigree information, and on the assumption that the frequency of the recessive allele in the cattle population from which the pedigree was sampled is equal to 0.00001, we calculate genotype probabilities for every member of the pedigree using the algorithm described above. Of the six founders present in this cattle pedigree, founder individual A2 is identified to be a carrier of the recessive allele with probability 1.0. Selective breeding decisions can be made given the calculated posterior genotype probabilities.

**Table 1:** Genetic profile of 26 individuals conditional on pedigree and phenotypic data.

				Genotype Probabilities			
				
Individual	Dam	Sire	Phenotype	Pr()	Pr()	Pr()	Pr()
A1, A4, A6	0	0	Normal	0.99999	0.000005	0.000005	0.0
A2	0	0	Normal	0.0	0.5	0.5	0.0
A3, A5	0	0	Normal	1.0	0.0	0.0	0.0
A7	A1	A2	Normal	0.0	1.0	0.0	0.0
A8	A3	A2	Normal	0.00001	0.99999	0.0	0.0
A9, A10, A11	A4	A2	Normal	0.0	0.99999	0.00001	0.0
A12, A13	A4	A8	Normal	0.0	0.99999	0.00001	0.0
A14	A5	A9	Normal	0.0	1.0	0.0	0.0
A15, A16	A6	A10	Normal	0.0	0.99999	0.00001	0.0
A17	A6	A10	Normal	0.5	0.5	0.0	0.0
A18	A6	A11	Normal	0.0	0.99999	0.00001	0.0
A19	A12	A9	Normal	0.33333	0.33333	0.33333	0.0
A20	A12	A9	Normal	0.33333	0.33333	0.33333	0.0
A21	A14	A15	Affected	0.0	0.0	0.0	1.0
A22	A14	A16	Affected	0.0	0.0	0.0	1.0
A23	A14	A7	Affected	0.0	0.0	0.0	1.0
A24, A25	A12	A9	Affected	0.0	0.0	0.0	1.0
A26	A13	A18	Affected	0.0	0.0	0.0	1.0

Pr() denotes the probability of an individual being homozygous for the recessive allele.

Next, we augment the genetic information used to calculate posterior genotype probabilities, by including genetic data on two marker loci flanking the hypothesized position of the recessive locus. Each marker locus has three alleles and the two loci are separated by 0.8 cM with the hypothesized position of the recessive locus 0.5 cM from the left marker (M1). The allele scores of the two markers used are given in Table 2. The impact of the additional information provided by the marker data is reflected in the posterior probability of individuals A19 and A20 being carriers of the recessive allele (Table 3). While without marker data individuals A19 and A20 have a posterior probability of being carriers equal to 0.6667, with marker data the probability is close to one.

**Table 2:** Marker allele scores for two markers flanking the causative recessive locus.

Individual	M1A1	M1A2	M2A1	M2A2
A1	1	1	3	1
A2	2	2	2	2
A3	3	3	2	2
A4	2	1	1	2
A5	3	1	2	1
A6	3	1	2	1
A7	2	1	2	1
A8	2	3	2	2
A9	2	1	2	1
A10	2	2	2	2
A11	0	0	0	0
A12	2	1	2	1
A13	0	0	0	0
A14	0	0	0	0
A15	2	1	2	1
A16	2	1	2	1
A17	2	3	2	2
A18	2	3	2	2
A19	2	1	2	1
A20	0	0	2	1
A21	2	2	2	2
A22	2	2	2	2
A23	2	2	2	2
A24	2	2	2	2
A25	2	2	2	2
A26	2	3	2	2

Each marker has three alleles coded as 1,2 and 3, with 0 denoting a missing value.

**Table 3:** Genetic profile of 26 individuals conditional on pedigree, marker and phenotypic data.

				Genotype Probabilities			
				
Individual	Dam	Sire	Phenotype	Pr()	Pr()	Pr()	Pr()
A1, A4, A6	0	0	Normal	1.0	0.0	0.0	0.0
A2	0	0	Normal	0.0	0.5	0.5	0.0
A3, A5	0	0	Normal	1.0	0.0	0.0	0.0
A7	A1	A2	Normal	0.0	1.0	0.0	0.0
A8	A3	A2	Normal	0.00001	0.99999	0.0	0.0
A9, A10, A11	A4	A2	Normal	0.0	0.99999	0.00001	0.0
A12, A13	A4	A8	Normal	0.0	1.0	0.0	0.0
A14	A5	A9	Normal	0.0	1.0	0.0	0.0
A15, A16	A6	A10	Normal	0.0	1.0	0.0	0.0
A17	A6	A10	Normal	0.49995	0.49995	0.00001	0.0
A18	A6	A11	Normal	0.0	0.99999	0.00001	0.0
A19	A12	A9	Normal	0.00003	0.49999	0.49999	0.0
A20	A12	A9	Normal	0.00299	0.4985	0.4985	0.0
A21	A14	A15	Affected	0.0	0.0	0.0	1.0
A22	A14	A16	Affected	0.0	0.0	0.0	1.0
A23	A14	A7	Affected	0.0	0.0	0.0	1.0
A24, A25	A12	A9	Affected	0.0	0.0	0.0	1.0
A26	A13	A18	Affected	0.0	0.0	0.0	1.0

Pr() denotes the probability of an individual being homozygous for the recessive allele.

## Discussion

As stated by Jensen and Kong [15] current algorithms for calculating marginal posterior genotype probabilities by peeling are inefficient. As described earlier, computing marginal genotype probabilities for individual *j *using equation 13, requires recomputing the likelihood for each of the possible genotypes of individual *j*. For the last individual in the peeling sequence, this can be done efficiently because intermediate results from peeling individuals 1 through *n *- 1, for each possible value of *g*_*n*_, have been stored in the anterior cutset . Thus, by making use of the intermediate results stored in , only calculations from the last step of peeling need to be repeated to compute . For any *m *that is in more than one set *V*_*i *_we identify the smallest *V*_*i *_containing *m*. The intermediate results from peeling individuals 1 through *i *are stored in anterior cutsets, including , and do not have to be recomputed. In this paper we have introduced a second type of cutset, called a posterior cutset, together with an algorithm for its efficient computation. The posterior cutset  contains the intermediate results from peeling all individuals that did not contribute to  and are not contained in the set *V*_*i*_. Thus, by making use of the intermediate results stored in both  and , only calculations associated with peeling individuals in *V*_*i *_(except *m*) need to be repeated to compute the numerator  of 13. For any *m *that is not in any *V*_*i *_the expression used to compute genotype probabilities (14) cannot be written as a product of a single anterior and posterior. However, any of the anterior the posterior cutsets used in 14 can be computed efficiently. Thus, this new peeling based algorithm provides an efficient method to compute marginal genotype probabilities for an arbitrary member of a pedigree with loops. The computational cost of obtaining posterior genotype probabilities for all members of a pedigree would approximately be equal to twice that of computing the likelihood because computing the likelihood only requires computing the anterior cutsets while computing all genotype probabilities would require computing the posterior cutsets also. As stated by Jensen and Kong [15], a peeling based algorithm would be more accessible to researchers in genetics than the currently available junction-tree methods [10].

Throughout this paper the likelihood was written as a sum over genotype variables. However, when the genotype of an individual is defined over *k *loci, the number of genotypes increases exponentially with *k*. In such situations, writing the likelihood as a sum over allele state and origin allele variables may lead to more efficient computations [12]. Algorithms presented in this paper can be used to calculate the posterior allele state and allele origin probabilities by peeling over allele state and allele origin variables.

## Competing interests

The authors declare that they have no competing interests.

## Authors' contributions

LRT and RLF developed and programmed the algorithm in C++. The analysis of the real cattle pedigree was performed by LRT. KJA contributed to the C++ implementation of the algorithm. The manuscript was prepared by LRT and RLF. All authors have read and approved the final manuscript.

## Appendix

**Figure 2:**
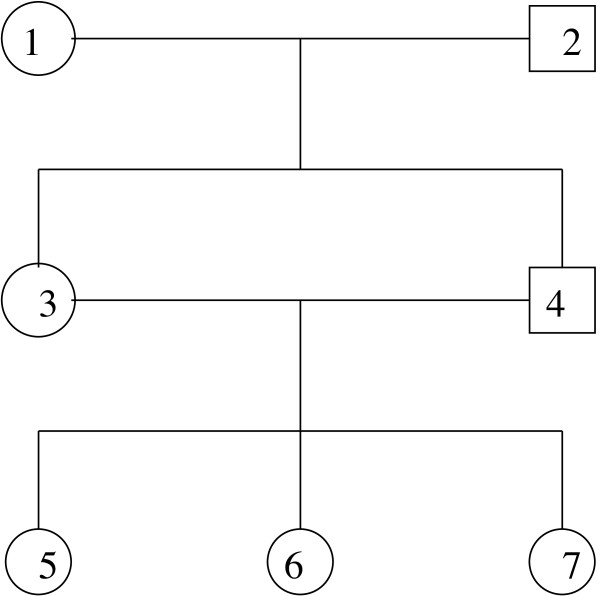
**Simple pedigree with loops**.

The pedigree given in Figure 2 will be used to illustrate the theoretical concepts discussed above.

First we show how to use the Elston-Stewart algorithm to compute the likelihood for a genetic model given this pedigree. Next we describe how to calculate marginal posterior genotype probabilities for an arbitrary member of this pedigree using the efficient algorithm described above.

### Likelihood computations by peeling

As shown in 2, the likelihood given the observed data can be written as(25)

In the pedigree given in Figure 2, individuals are numbered according to a suitable peeling sequence. Note that in 25 *f*_1_(*g*_5_, *g*_4_, *g*_1_) is the only function that involves *g*_1_. Peeling *g*_1 _amounts to computing the sum over *g*_1 _of *f*_1_(*g*_5_, *g*_4_, *g*_1_), for each combination of the genotypes for individuals 5 and 4, and storing the results of these summations in the anterior cutset

Note that  is a two dimensional table of size *N*_5 _× *N*_4_, where *N*_5 _and *N*_4 _are the number of possible genotypes for individuals 5 and 4. Replacing the sum over *g*_1 _of *f*_1_(*g*_5_, *g*_4_, *g*_1_) in 25 with  gives the likelihood expression *LE*_1_:

Note that in *LE*_1 _*f*_2_(*g*_5_, *g*_4_, *g*_2_) is the only function that involves *g*_2_. Therefore, the anterior cutset for 2 (obtained by peeling *g*_2_) is

Replacing the sum over *g*_2 _of *f*_2_(*g*_5_, *g*_4_, *g*_2_) in *LE*_1 _with  gives the likelihood expression *LE*_2_:

Note that in *LE*_2 _*f*_3_(*g*_5_, *g*_4_, *g*_3_) is the only function that involves *g*_3_. Therefore, the anterior cutset for 3 (obtained by peeling *g*_3_) is(26)

Replacing the sum over *g*_3 _of *f*_3_(*g*_5_, *g*_4_, *g*_3_) in *LE*_2 _with  gives the likelihood expression *LE*_3_:

Note that in *LE*_3 _not only *f*_4_(*g*_7_, *g*_6_, *g*_4_), but also ,  and  involve *g*_4_. Thus, peeling *g*_4 _yields the following anterior cutset(27)

The resulting anterior cutset  is a three dimensional table of size *N*_7 _× *N*_6 _× *N*_5_, where *N*_7_, *N*_6 _and *N*_5 _are the number of possible genotypes for individuals 7, 6 and 5.  replaces in *LE*_3 _the factors *f*_4_(*g*_7_, *g*_6_, *g*_4_), ,  and  summed over *g*_4_. Thus, the likelihood expression *LE*_4 _becomes

Note that in *LE*_4 _both *f*_5_(*g*_7_, *g*_6_, *g*_5_) and  involve *g*_5_. Peeling *g*_5 _yields the following anterior cutset(28)

This cutset replaces in *LE*_4 _the factors *f*_5_(*g*_7_, *g*_6_, *g*_5_) and  summed over *g*_5_. Thus, the likelihood expression *LE*_5 _becomes

In *LE*_5 _both *f*_6_(*g*_6_) and  involve *g*_6_. Peeling *g*_6 _yields the following anterior cutset(29)

By replacing *f*_6_(*g*_6_) and  summed over *g*_6 _with  in *LE*_5_, the likelihood expression *LE*_6 _becomes

Note, however, that the anterior cutset obtained by peeling *g*_7 _yields the numerical value(30)

and thus the likelihood expression *LE*_7_:

### Genotype probability computations

Recall that for an arbitrary member of the pedigree (e.g. individual 3) we can calculate marginal genotype probabilities as follows(31)

where  is the likelihood computed with *g*_3 _fixed at *x*. As discussed earlier, using this procedure to compute marginal genotype probabilities for *N *unknown genotypes of individual 3 requires recomputing the likelihood for the entire pedigree *N *times. However by writing the likelihood as in 12, these computations can be done efficiently. Consider computing marginal posterior genotype probabilities for individual 3. Recall that, as shown in 26,  = Σ_*g*3 _*f*_3_(*g*_5_, *g*_4_, *g*_3_). Using this in 12 we obtain(32)

Note that 32 can be used to calculate the denominator of 31, while the numerator of 31 can be obtained by fixing *g*_3 _in 32 at *x*. To complete the calculations, however, we need to compute . This is done using the recursive procedure described previously as shown below.

Step 1 of the procedure is to compute anterior cutsets for all individuals in the pedigree, and this has already been done. Following step 2, we determine that  contributes to the computation of  (see equation 27). Following step 3,  is replaced with  in 27 and, for each value of *g*_4 _and *g*_5_, the sum over *g*_7 _and *g*_6 _is computed to obtain(33)

Following step 4, note that  is not computed yet. Thus, steps 2, 3 and 4 are repeated as follows.

Following step 2, we determine that  contributes to the computation of  (see equation 28). Following step 3,  is replaced with  in 28 and, for each value of *g*_7_, *g*_6 _and *g*_5_, we obtain(34)

Following step 4, note that  is not computed yet. Thus, steps 2, 3 and 4 are repeated as follows.

Following step 2, we determine that  contributes to the computation of  (see equation 29).

Following step 3,  is replaced with  in 29 and, for each value of *g*_7 _and *g*_6 _we obtain

Following step 4, note that  is not computed yet. Thus, steps 2, 3 and 4 are repeated as follows.

Following step 2, we determine that  contributes to the computation of  (see equation 30).

Following step 3,  is replaced with  in 30 and, for each value of *g*_7 _we obtain

Following step 4, note that  = 1.0, and thus the calculations for  can be completed. Now using , the calculations for  can be completed, and using , the calculations for  can be completed. Finally, using , the calculations for  can be completed.

## Supplementary Material

Additional file 1 **A numerical example to illustrate algorithm to detect loops in a pedigree**. Algorithm to detect loops in a pedigree. Click here for file
