# Supplementary material for: An efficient algorithm to compute marginal posterior genotype probabilities for every member of a pedigree with loops
Source: Genet Sel Evol. 2009 Dec 3;41(1):52. doi: 10.1186/1297-9686-41-52 (PMC2801663; doi:10.1186/1297-9686-41-52)
Supplement: Additional file 1 — A numerical example to illustrate algorithm to detect loops in a pedigree. Algorithm to detect loops in a pedigree. [file 1297-9686-41-52-S1.PDF]

Algorithm to detect loops in a pedigree

L. Radu Totir[1]and Rohan L. Fernando*[2]

[1]Pioneer Hi-Bred, A Dupont Company, 7250 NW 62nd Ave., Johnston, Iowa, 50131-0552, USA
[2]Department of Animal Science and Center for Integrated Animal Genomics, Iowa State University, Ames, IA 50011, USA

Email: L. Radu Totir - radu.totir@pioneer.com; Rohan L. Fernando*- rohan@iastate.edu;

*Corresponding author

A pedigree is a set of individuals, each of which can be classified as a founder or a non-founder. A founder is a pedigree member whose parents are not in the pedigree, and a non-founder is a pedigree member with one or both parents included in the pedigree. The algorithm used to determine if a pedigree contains loops relies on identifying and then eliminating terminal members from the pedigree. Terminal members of a pedigree are members of terminal families that do not belong to another family. A terminal family is a family that has at most one member who belongs to at least one other nuclear family. A nuclear family consists of a set of parents and all their offspring. If a pedigree does not contain any loops, repeated removal of terminal members from the pedigree will result in all members being removed from the pedigree. On the other hand, if a pedigree contains any loops, not all members of the pedigree can be removed by repeated removal of terminal members.

Consider first the pedigree given in Figure 1. To apply the algorithm described above, first remove the

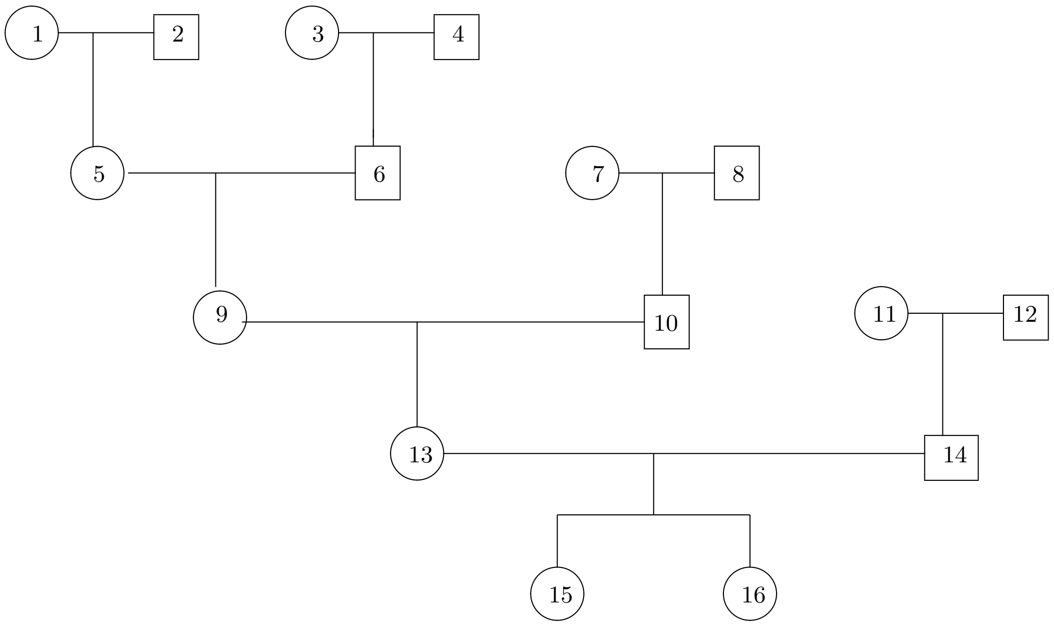

Figure 1: Original pedigree with no loops

terminal members 1,2,3,4,7,8,11 and 12 from the terminal families made up of (1,2,5), (3,4,6), (7,8,10) and

(11,12,14). After removing these terminal members, we obtain the modified pedigree given in Figure 2. Next, in the pedigree given in Figure 2, remove the terminal members 5,6,14,15 and 16 from their respective

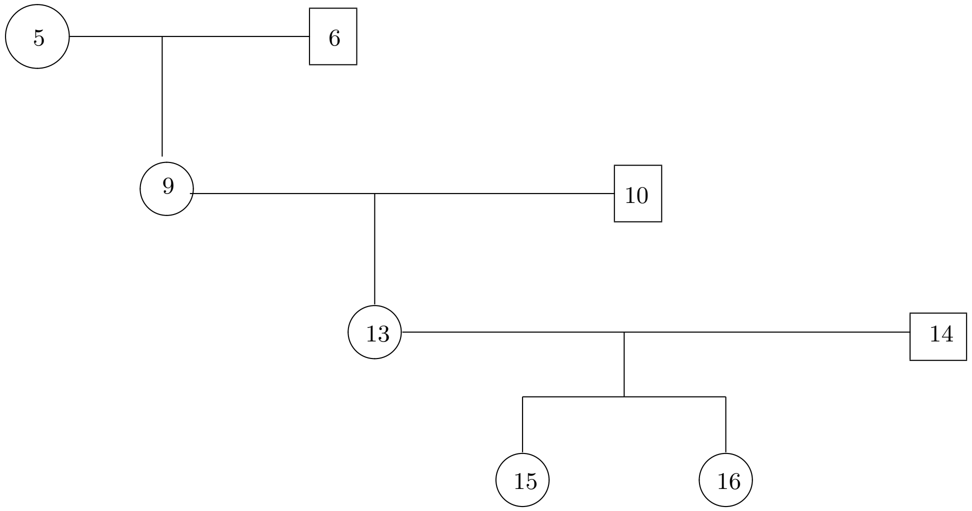

Figure 2: Modified pedigree with no loops

terminal families (5,6,9) and (13,14,15,16). Finally we are left with one terminal family (9,10,13) in which all family members are terminal and can be removed. Because repeated removal of terminal members from the pedigree given in Figure 1 results in all members being removed from the pedigree, we conclude that this pedigree does not have loops.

Next we apply the same algorithm to the pedigree given in Figure 3. First remove the terminal members 12 and 13 from the terminal family (11,12,13). Once 12 and 13 have been removed from the pedigree, members 10 and 11 become terminal and are removed from the terminal family (9,10,11). Consequently, members 8 and 9 become terminal members and are removed from the terminal family (7,8,9). After successive removals of these terminal members, we obtain the pedigree given in Figure 4. This pedigree contains two nuclear families: (1,2,3,4) and (3,4,5,6,7), neither of which is terminal. Because repeated removal of terminal members from the pedigree given in Figure 3 does not result in all members being removed from the pedigree, we conclude that this pedigree does have loops.

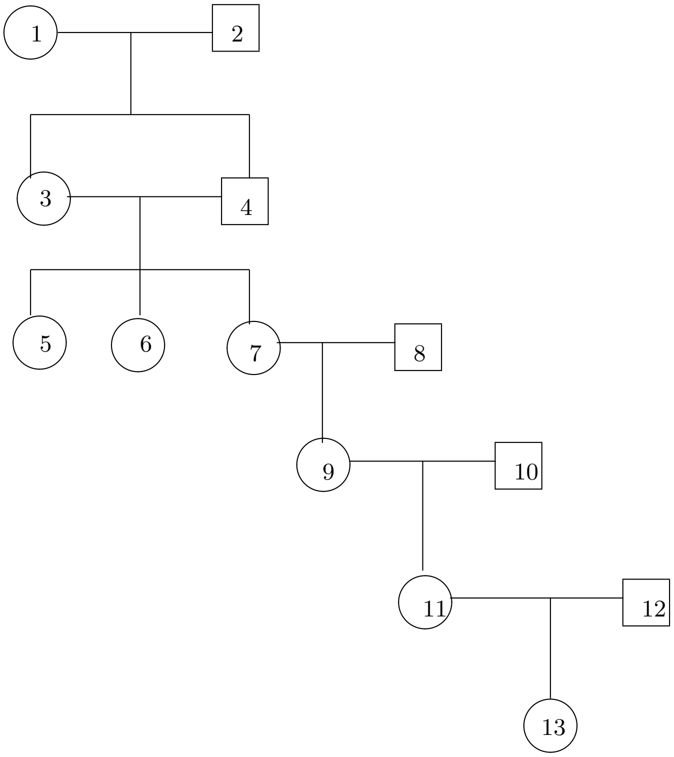

Figure 3: Original pedigree with loops

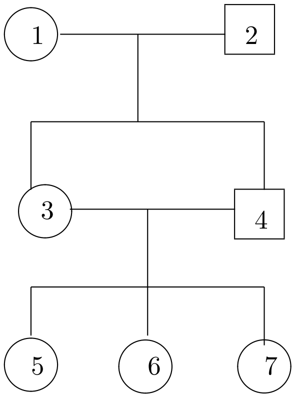

Figure 4: Modified pedigree with loops
